# Hydroimidazolone Modification of the Conserved Arg12 in Small Heat Shock Proteins: Studies on the Structure and Chaperone Function Using Mutant Mimics

**DOI:** 10.1371/journal.pone.0030257

**Published:** 2012-01-17

**Authors:** Ram H. Nagaraj, Alok Kumar Panda, Shilpa Shanthakumar, Puttur Santhoshkumar, NagaRekha Pasupuleti, Benlian Wang, Ashis Biswas

**Affiliations:** 1 Department of Ophthalmology and Visual Sciences, Case Western Reserve University, Cleveland, Ohio, United States of America; 2 School of Basic Sciences, Indian Institute of Technology Bhubaneswar, Orissa, India; 3 Department of Ophthalmology, University of Missouri-Columbia, Columbia, Missouri, United States of America; 4 Center for Proteomics and Bioinformatics, Case Western Reserve University, Cleveland, Ohio, United States of America; University of Arkansas for Medical Sciences, United States of America

## Abstract

Methylglyoxal (MGO) is an α-dicarbonyl compound present ubiquitously in the human body. MGO reacts with arginine residues in proteins and forms adducts such as hydroimidazolone and argpyrimidine *in vivo*. Previously, we showed that MGO-mediated modification of αA-crystallin increased its chaperone function. We identified MGO-modified arginine residues in αA-crystallin and found that replacing such arginine residues with alanine residues mimicked the effects of MGO on the chaperone function. Arginine 12 (R12) is a conserved amino acid residue in Hsp27 as well as αA- and αB-crystallin. When treated with MGO at or near physiological concentrations (2–10 µM), R12 was modified to hydroimidazolone in all three small heat shock proteins. In this study, we determined the effect of arginine substitution with alanine at position 12 (R12A to mimic MGO modification) on the structure and chaperone function of these proteins. Among the three proteins, the R12A mutation improved the chaperone function of only αA-crystallin. This enhancement in the chaperone function was accompanied by subtle changes in the tertiary structure, which increased the thermodynamic stability of αA-crystallin. This mutation induced the exposure of additional client protein binding sites on αA-crystallin. Altogether, our data suggest that MGO-modification of the conserved R12 in αA-crystallin to hydroimidazolone may play an important role in reducing protein aggregation in the lens during aging and cataract formation.

## Introduction

Small heat shock proteins are a family of stress proteins. α-Crystallin and Hsp27 are the major small heat shock proteins in humans. These proteins are beneficial in preventing cellular damage for various diseases [1], [2], [3].

α-Crystallin is a major protein of vertebrate eye lenses, although its presence in other organs such as the brain, heart, kidney, spleen and thymus has also been recognized [4], [5]. α-Crystallin consists of two highly homologous subunits, αA- and αB-crystallin, and each subunit has a molecular weight of ∼20 kDa [4]. In the lens αA-crystallin and αB-crystallin subunits combine in a 3∶1 ratio to form an ∼40 mer α-crystallin oligomer [6]. The αB-crystallin gene has a heat shock promoter element and is induced by various stress conditions [4], [5], [7]. αB-Crystallin has been implicated in a number of neurological disorders, such as Alzheimer's disease and Parkinson's disease [1], [8]. Both αA-crystallin and αB-crystallin can confer cellular thermo-resistance [9]. Both proteins can act as molecular chaperones, and this chaperoning ability is believed to play a crucial role in maintaining the transparency of the eye lens [10]. As a molecular chaperone, α-crystallin not only prevents the aggregation of unfolded proteins, but it also helps in the refolding of denatured client proteins [11], [12]. Because protein turnover is virtually absent in the lens, many post-translational modifications accumulate in lens proteins during aging. Several studies have shown that these post-translational modifications decrease the chaperone function of α-crystallin, which might be one reason for lens aging and age-related cataract formation [13], [14], [15], [16], [17].

A large number of advanced glycation end products (AGEs) can be found in the aged human lens [18], which suggests that glycation is a major mechanism for post-translational modification in the aging lens. Glycation is the non-enzymatic reaction that adds carbohydrates, especially glucose, to proteins. First, glucose and other sugars react with the amino groups of proteins to form an unstable Schiff's base that slowly undergoes rearrangement to form a relatively stable Amadori product. Through a series of parallel and sequential reactions (often termed the Maillard reaction), these Amadori products form many AGEs, some of which are fluorescent and colored [19], [20].

**Figure 1:**
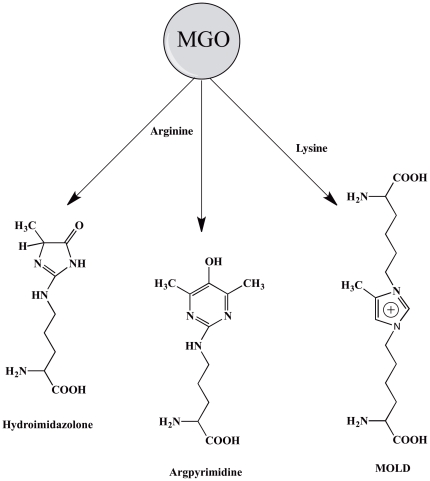
MGO reacts with proteins to form AGEs, like, hydroimidazolone, argpyrimidine and MOLD in tissue proteins.

The lens contains relatively high levels of methylglyoxal (MGO). The reported levels are 1–2 µM [21]. MGO is an α-dicarbonyl compound that reacts with lysine, arginine and histidine residues in proteins [22], [23] to form AGEs, such as hydroimidazolone [24], argpyrimidine [25] and methylglyoxal lysine dimer (MOLD) [26], [27] (Fig. 1). In addition to our own previous findings, others have reported that the aged and cataractous human lenses contain more of these MGO-derived AGEs than the normal lens [25], [27], [28], [29]. Because MGO reacts rapidly with proteins and the lens proteins have long half-lives, it is reasonable to assume that cumulative modification by MGO over many decades of life could be quantitatively significant in the lens proteins.

In general, it is believed that AGE formation is a cause for lens protein aging and cataract formation. However, we and others have observed that MGO-AGE formation in αA-crystallin makes it a better chaperone [30], [31]. AGE formation from MGO occurs predominantly in arginine residues of proteins. Examples of arginine-derived AGEs caused by MGO glycation are argpyrimidine and hydroimidazolone [18]. As a result of these modifications, arginine residues lose their positive charge and become neutral. In a previous study, we demonstrated that the loss of the positive charge was the cause for an increase in the chaperone function of αA-crystallin. In that study, we replaced discrete MGO-modifiable arginine residues with a neutral amino acid, alanine, and showed an improvement in the chaperone function of the mutant proteins [32]. In addition, the chemical conversion of lysine residues to homoarginine residues followed by a reaction with MGO also led to an enhancement in the chaperone function of αA-crystallin [33].

Unlike α-crystallin, Hsp27 is ubiquitously expressed throughout the human body. We have shown that Hsp27 is particularly vulnerable to MGO modification in kidney mesangial cells [34]. Others have shown a similar vulnerability of Hsp27 in other cell types [35], [36]. Furthermore, we showed that the chaperone and anti-apoptotic functions of Hsp27 were improved after its modification by MGO [37]. Thus, Hsp27 appears to be a prime target for MGO modification, and consequently, its function could be altered in cells.

Altogether, it is clear now that MGO modification of the small heat shock proteins results in an improvement in their key functions. Whether the improvement in the chaperone function of small heat shock proteins occurs via modification of a conserved arginine residue and whether physiological levels of MGO could improve the chaperone function through a hydroimidazolone modification is not known. In this study, we modified human Hsp27 and αA- and αB-crystallin with 2–10 µM MGO and identified hydroimidazolone AGEs using mass spectrometry. Interestingly, the only conserved arginine residue that was modified to hydroimidazolone by MGO was R12 in all three proteins. To determine if the hydroimidazolone modification of this arginine residue is responsible for the improvement of the chaperone function, we replaced R12 with alanine (to mimic the hydroimidazolone modification) and explored the effect of this mutation on the structure and chaperone function of Hsp27 and αA- and αB-crystallin.

## Results and Discussion

**Figure 2:**
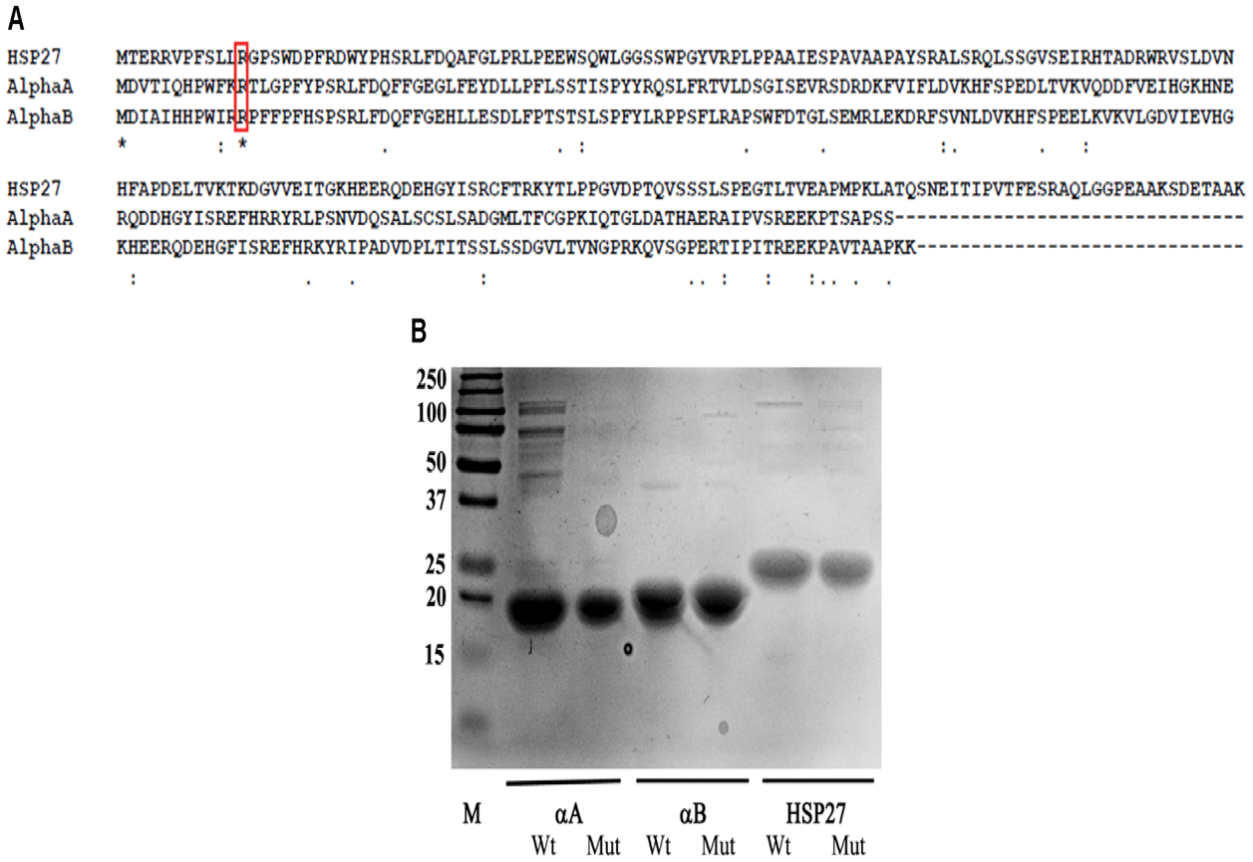
Sequence alignment and SDS-PAGE of recombinant human Hsp27, αA- and αB-crystallin. (**A**) Amino-acid sequence alignment between these three small heat shock proteins was performed using the MULTIPLE SEQUENCE ALIGNMENT program (T-Coffee). (**B**) SDS-PAGE of purified proteins. M = Molecular weight markers.

MGO is derived mostly from triose phosphate intermediates of glycolysis by non-enzymatic mechanisms *in vivo* [38]. It is a major precursor of AGEs in tissue proteins [39]. In previous studies, we have shown that MGO modifications of small heat shock proteins, such as αA-crystallin and Hsp27, enhanced their chaperone function [30], [37]. In this study, our primary goal was to determine whether a similar increase in the chaperone function occurred with physiological levels of MGO and to determine whether a modification of the conserved R12 (Fig. 2A) to hydroimidazolone contributed to the increased chaperone function.

We first determined the “first hit” arginine residues for modification to hydroimidazolone. To accomplish this, we modified the proteins with 2, 5 and 10 µM of MGO. With 2 µM MGO, we found that 6, 6 and 8 arginine residues were modified to hydroimidazolone in αA- and αB-crystallin and Hsp27, respectively (Table 1). With 10 µM of MGO, this modification reached 10, 8 and 10 arginine residues in the three respective proteins. R12 was the only common residues among the three proteins converted to hydroimidazolone with 2 µM MGO, which suggested that in small heat shock proteins, R12 is the most susceptible for modification to hydroimidazolone by MGO. Notably, a previous study detected a modification of R12 in human lens αA-crystallin that had a molecular weight identical to hydroimidazolone [40].

**Table 1:** Identification of HI modification with the treatment of MGO detected by LC-MS/MS.

Protein	Peptide	Mass (obs.)	Mass (cal.)	Modified Arg residues	Concentration of MGO (µM)		
					2	5	10
αA-crystallin	**R**TLGPFYPSR	1246.6462	1246.6458	R12	X	X	X
	QSLF**R**TVLDSGISEVR	1859.9809	1859.9741	R54			X
	TVLDSGISEV**R**SDRDK	1829.9117	1829.9119	R65	X	X	X
	SD**R**DKFVIFLDVK	1634.8756	1634.8668	R68	X	X	X
	HNE**R**QDDHGYISR	1679.7469	1679.7400	R103		X	X
	QDDHGYIS**R**EFHR	1712.7664	1712.7655	R113			X
	**R**YRLPSNVDQSALSCSLSADGMLTFCGPK	3283.5509	3283.5424	R117			X
	Y**R**LPSNVDQSALSCSLSADGMLTFCGPK	3143.4382	3143.4362	R119	X	X	X
	IQTGLDATHAE**R**AIPVSR	1988.0434	1988.0439	R157	X	X	X
	AIPVS**R**EEKPTSAPSS	1708.8624	1708.8631	R163	X	X	X
αB-crystallin	**R**PFFPFHSPSR	1427.7094	1427.7099	R12	X	X	X
	APSWFDTGLSEM**R**LEK	1935.9078	1935.9036	R69	X	X	X
	LEKD**R**FSVNLDVK	1615.8580	1615.8569	R74	X	X	X
	HEE**R**QDEHGFISR	1692.7621	1692.7604	R107	X	X	X
	HEERQDEHGFIS**R**EFHR	2262.0316	2262.0314	R116		X	X
	Y**R**IPADVDPLTITSSLSSDGVLTVNGPR	2996.5399	2996.5455	R123		X	X
	KQVSGPE**R**TIPITR	1634.9041	1634.9104	R157	X	X	X
	TIPIT**R**EEKPAVTAAPK	1875.0456	1875.0465	R163	X	X	X
Hsp27	**R**VPFSLLR	1040.6157	1040.6131	R5	X	X	X
	VPFSLL**R**GPSWDPFR	1826.9545	1826.9468	R12	X	X	X
	GPSWDPF**R**DWYPHSR	1955.8783	1955.8703	R20	X	X	X
	DWYPHS**R**LFDQAFGLPR	2158.0497	2158.0385	R27	X	X	X
	ALS**R**QLSSGVSEIR	1555.8312	1555.8318	R79	X	X	X
	QLSSGVSEI**R**HTADR	1708.8496	1708.8492	R89	X	X	X
	QLSSGVSEIRHTAD**R**WR	2051.0302	2051.0297	R94		X	X
	W**R**VSLDVNHFAPDELTVK	2179.1149	2179.1062	R96	X	X	X
	TKDGVVEITGKHEE**R**QDEHGYISR	2836.3729	2836.3740	R127			X
	QDEHGYIS**R**CFTR	1721.7586	1721.7580	R136	X	X	X

The modification of arginine residues to hydroimidazolone converts the positive charge on arginine to a neutral charge. Previously, we reported that the substitution of MGO-modifiable arginine residues with neutral alanine residues enhanced the chaperone function of αA-crystallin, similarly to MGO-modification [32]. Because R12 is the most susceptible arginine for MGO modification, we sought to determine if the chaperone function would be improved if it was replaced with alanine. To accomplish this, we cloned and expressed the wild-type (Wt) proteins and the Hsp27_R12A_, αA-crystallin_R12A_ (αA_R12A_) and αB-crystallin_R12A_ (αB_R12A_) mutant proteins in *E. coli* BL21(DE3). We then purified the proteins by sequential chromatographic methods (gel filtration and ion-exchange chromatography), as previously described [41]. SDS-PAGE analysis showed a single protein band with the correct molecular weight for all proteins (Fig. 2B).

**Figure 3:**
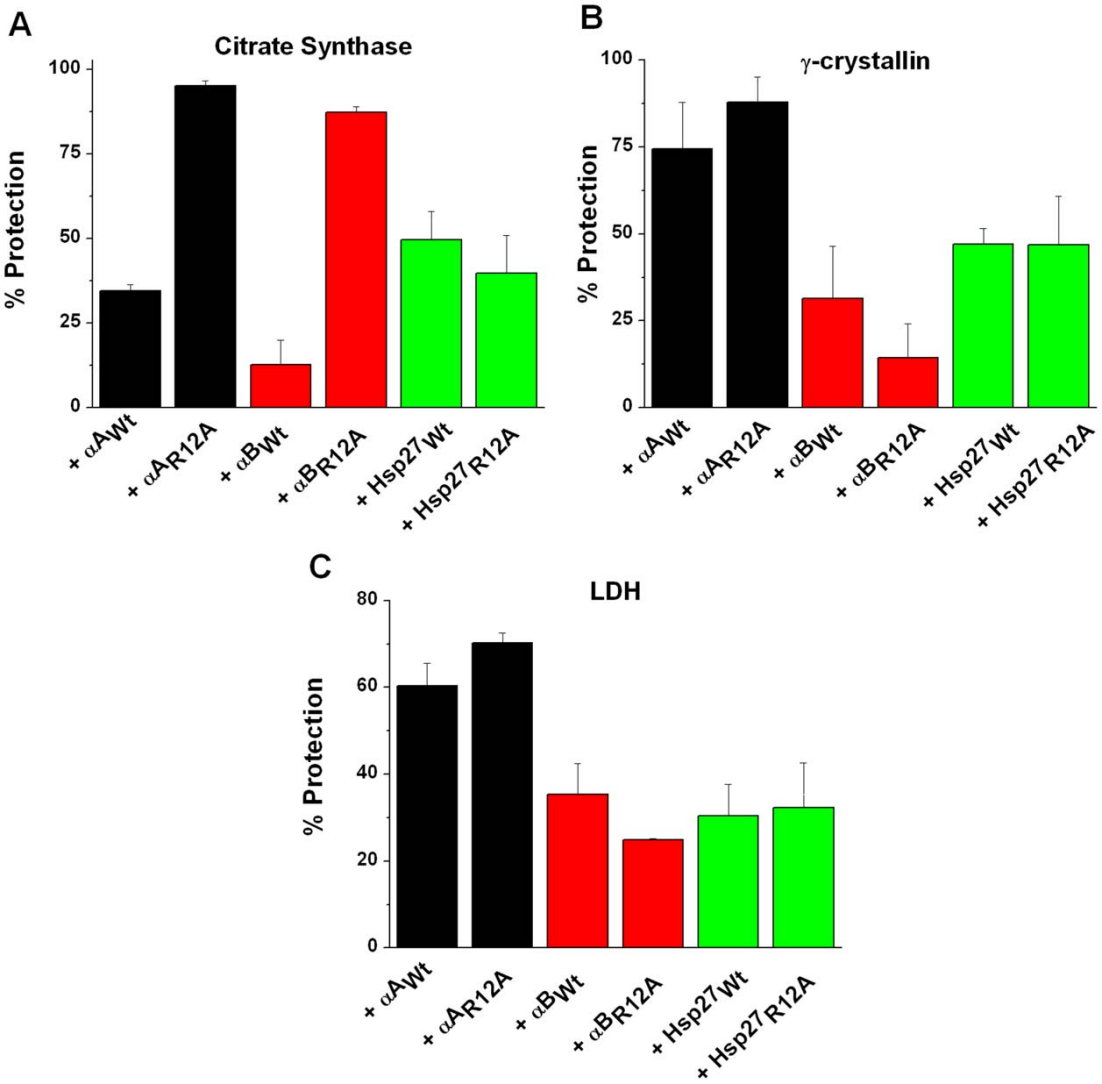
Effect of R12A mutation on the chaperone function of Hsp27, αA- and αB-crystallin. The chaperone function of these three small heat shock proteins (wild type and mutants)was assessed using three client proteins, as described in *Materials and Methods*. (**A**) Citrate synthase (CS); (**B**) γ-crystallin and (**C**) Lactate dehydrogenase (LDH).

The chaperone function for the small heat shock proteins was evaluated using three different client proteins. The αA_R12A_ mutant showed a 61%, 15% and 10% increase in the chaperone function compared to the Wt protein with CS, γ-crystallin and LDH, respectively, as client proteins (Fig. 3). Although, αB_R12A_ showed better protection against thermal aggregation of CS than its Wt variant (∼70% better protective ability), it showed a slight reduction in the chaperone function against the other two client proteins tested (Fig. 3B & C). The R12A mutation had no effect on the chaperone function of Hsp27 (Fig. 3A–C). Previously, Oya-Ito *et al.* [37] showed that MGO modification made Hsp27 a better chaperone. The findings in this study that theR12A mutation did not improve the chaperone function of Hsp27 suggest that in addition to hydroimidazolone modification, argpyrimidine modification may be necessary for the improvement of the chaperone function inHsp27.

**Figure 4:**
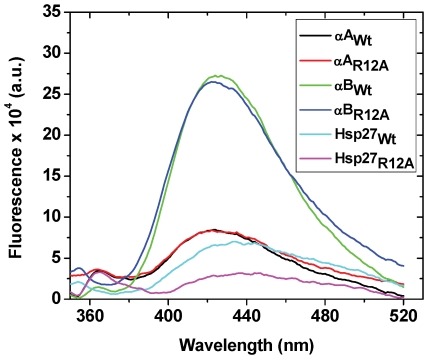
Effect of R12A mutation on the surface hydrophobicity of Hsp27 and α-crystallin. The surface hydrophobicity of wild type and mutant proteins was estimated using a hydrophobic probe, TNS. Protein concentration was 0.1 mg/ml and TNS concentration was 100 µM. The fluorescence spectrum of TNS bound to different samples at 25°C was recorded from 350–520 nm. The excitation wavelength was 320 nm.

To understand the molecular basis behind the enhancement in the chaperone function of αA_R12A_, we determined the structural changes in the protein. Numerous studies have suggested a strong correlation between the chaperone function and the surface hydrophobicity of small heat shock proteins [42], [43], [44], [45], [46], [47], but others have failed to find such a correlation [48], [49]. The surface hydrophobicity of αA_R12A_ and αB_R12A_ (determined by TNS binding) were nearly identical to that of their Wt protein counterparts. Hsp27_R12A_ had a decreased (∼65%) surface hydrophobicity compared to its Wt counterpart (Fig. 4). These results suggested a lack of correlation between surface hydrophobicity and the chaperone function in the three small heat shock proteins, which is similar to previous reports [48], [49].

**Figure 5:**
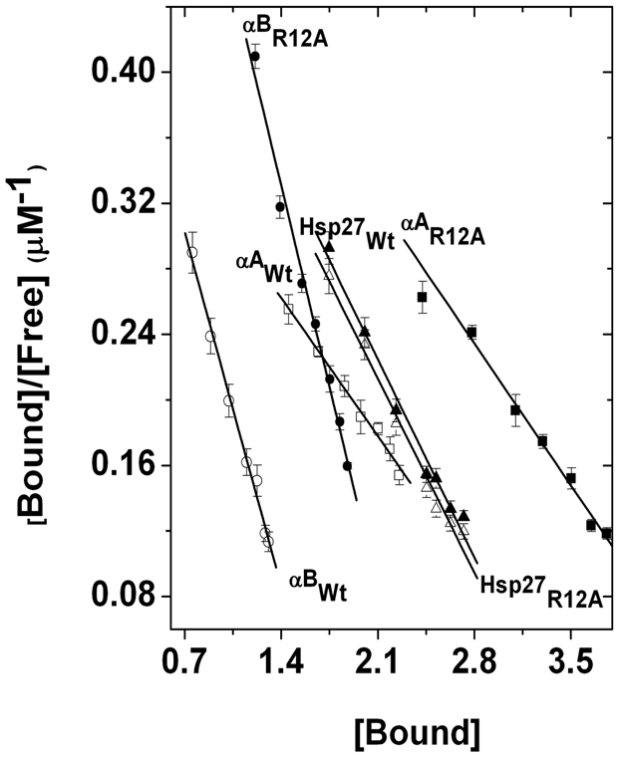
Binding constant of wild type and R12A mutants of Hsp27, αA- and αB-crystallin for γ-crystallin. Binding parameters for the interaction between γ-crystallin and different small heat shock proteins at 60°C were estimated from Scatchard plot.

To assess whether the binding sites of these heat shock proteins for client proteins were altered by the R12A mutation, we performed an equilibrium binding study using γ-crystallin as the client protein. We incubated the three heat shock proteins (12.5 µM each) for 1 hr at 60°C with various concentrations of γ-crystallin (2–18 µM). The unbound (S) and bound γ-crystallin were determined by filtration, as described in *Materials and Methods* section. We determined the dissociation constant (K_d_) using the Scatchard equation:
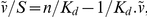
where 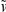 is the number of moles of the substrate bound per mole of chaperone, and *n* is the number of binding site and K_d_ is the dissociation constant. The stoichiometry of *n* and K_d_ obtained from the Scatchard plot (Fig. 5) is 3.61 per subunit of αA-crystallin and 8.44 µM, respectively (Table 2). We noted that the number of binding sites (*n*) per subunit of αA_R12A_ increased from 3.61 in Wt to 4.68, and the association constant increased from 0.118 µM^−1^ (K_d_ = 8.44 µM) in Wt to 0.124 µM^−1^ (K_d_ = 8.07 µM) in αA_R12A_. The *n* and K_d_ values decreased ∼25% and ∼13%, respectively, in αB_R12A_. In contrast, Hsp27_R12A_ showed no changes in either of these parameters when compared to its Wt counterpart (Table. 2). From these data, we concluded that the substitution of alanine for the conserved arginine residue at position 12 of αA-crystallin increased its affinity for denatured client proteins, whereas the same substitution in αB-crystallin lowered its interaction with the denatured substrate protein. Our results also demonstrated that TNS binding sites are different than the client protein binding sites in all three proteins. We speculate that a structural alteration in αA_R12A_ exposed additional client protein binding sites, and thus, αA_R12A_ bound more client proteins and exhibited better chaperone function than its Wt counterpart.

**Table 2:** Determination of the number of binding sites (n) and dissociation constant (K_d_) values for the interaction of human Hsp27 and αA-and αB-crystallin and their R12A mutants with γ-crystallin at 60°C.

System studied	n	K_d_ (µM)
αA_Wt_+γ-crystallin	3.61±0.07	8.44±0.42
αA_R12A_+γ-crystallin	4.68±0.09	8.07±0.28
αB_Wt_+γ-crystallin	2.34±0.11	2.84±0.12
αB_R12A_+γ-crystallin	1.68±0.07	3.24±0.17
Hsp27_Wt_+γ-crystallin	2.90±0.08	5.91±0.23
Hsp27_R12A_+γ-crystallin	2.87±0.13	5.79±0.35

**Figure 6:**
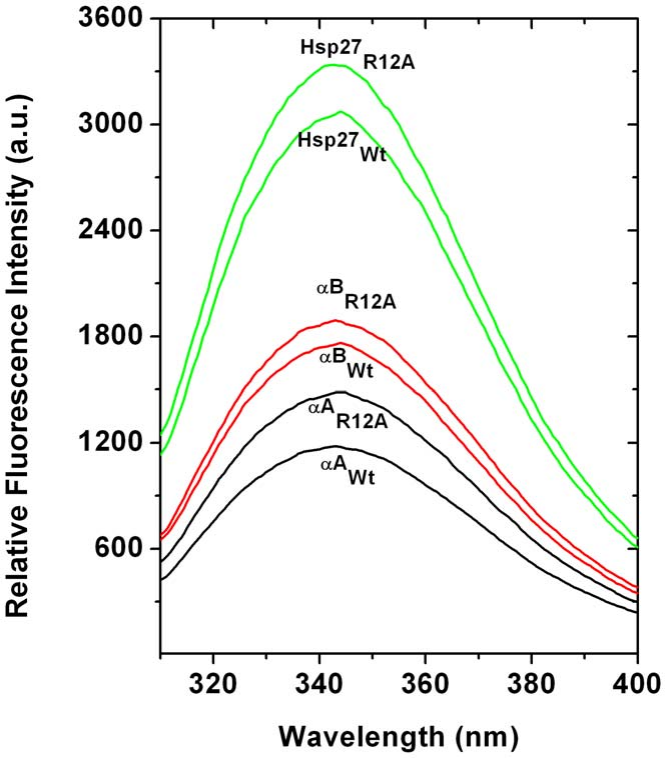
Intrinsic tryptophan fluorescence spectra of wild type and mutant (R12A) Hsp27, αA- and αB-crystallin. Tryptophan fluorescence spectra of different samples (0.1 mg/ml protein) were recorded from 310–400 nm at 25°C. The excitation wavelength was 295 nm. Data were collected at 0.5 nm wavelength resolution.

We used tryptophan (W) fluorescence along with near- and far-UV CD techniques to determine if there were any changes in the tertiary and secondary structures of the Wt proteins compared to the mutant proteins. The intrinsic fluorescence spectra indicated some differences between Wt and mutant proteins (Fig. 6). The fluorescence intensity of αA_R12A_, αB_R12A_ and Hsp27_R12A_ increased ∼27%, 8% and 10%, respectively, compared to the corresponding Wt proteins. Moreover, the λ_max_ of the tryptophan fluorescence spectra of the wild-type proteins did not alter due to the mutation. The changes in fluorescence intensity may reflect changes in the microenvironment of W9 (in αA- and αB-crystallin) and W16 (in Hsp27), which are located close to the mutation sites. The near-UV CD spectra of these three proteins (both wild type and mutant) agreed with our intrinsic fluorescence data (data not shown). However, these changes in tryptophan fluorescence (perturbation in tertiary structure) did not correlate with the changes in the chaperone function. While some studies showed a direct relationship between an increase in tryptophan fluorescence with improved chaperone function, others did not find such a relationship [33], [50], [51], [52]. Therefore, it is unclear whether changes in the microenvironment of tryptophan are determinants of changes in the chaperone function of α-crystallin.

Quantitative analysis of the far-UV CD data using the CONTINLL program showed that Hsp27 and αA- and αB-crystallin are major β-sheet proteins (Table 3). The data showed no significant perturbation in the secondary structure in the three proteins as a result of the R12A mutation. Based on these data, we concluded that the contribution of R12 for the secondary structure in the three small heat shock proteins was minimal.

**Table 3:** Percent levels of secondary structure in the wild-type and R12A mutants of human Hsp27 and αA- and αB-crystallin using CONTINLL software.

Protein	α-helix	β-sheet	β-Turn	Random
αA_Wt_	3.97	30.77	24.83	39.33
αA_R12A_	3.30	33.27	23.73	38.80
αB_Wt_	3.87	36.73	22.73	35.87
αB_R12A_	1.83	30.17	25.23	42.13
Hsp27_Wt_	3.07	39.63	20.97	35.33
Hsp27_R12A_	3.57	34.60	23.43	38.87

Multi-angle light scattering experiments determine the polydispersity and the absolute molar mass of proteins. We used this technique to determine whether the subtle changes in tertiary structure altered the quaternary structure (*i.e.*, the oligomeric assembly) of these three small heat shock proteins. From the data in Table 4, it is evident that a perturbation in the tertiary structure had little effect on the molecular mass of the mutant proteins. The hydrodynamic radius (R_h_) was slightly increased only with αA_R12A_ (Table 4). Kundu *et al.* [53] previously reported that the deletion of the first 20 amino acid residues in αA-crystallin had no effect in its oligomeric size, which is analogous to the findings in this study. The relationship between oligomeric size and chaperone function of small heat shock proteins is still unclear. Some studies have shown that higher oligomeric assembly diminishes the chaperone function of these proteins [52], [54], whereas others have demonstrated contrary results [55], [56]. Our results failed to find a correlation between oligomeric size and chaperone function of these three small heat shock proteins.

**Table 4:** The molar mass and the hydrodynamic radius of the wild-type and R12A mutants of α-crystallin and Hsp27.

Proteins	Molar Mass (g/mol)	Hydrodynamic radius (nm)
αA_Wt_	(4.787±0.003) e+5	6.94±0.18
αA_R12A_	(5.491±0.005) e+5	7.65±0.21
αB_Wt_	(4.804±0.005) e+5	6.59±0.17
αB_R12A_	(4.316±0.003) e+5	6.52±0.17
Hsp27_Wt_	(6.085±0.004) e+5	7.71±0.22
Hsp27_R12A_	(5.010±0.006) e+5	7.60±0.26

**Figure 7:**
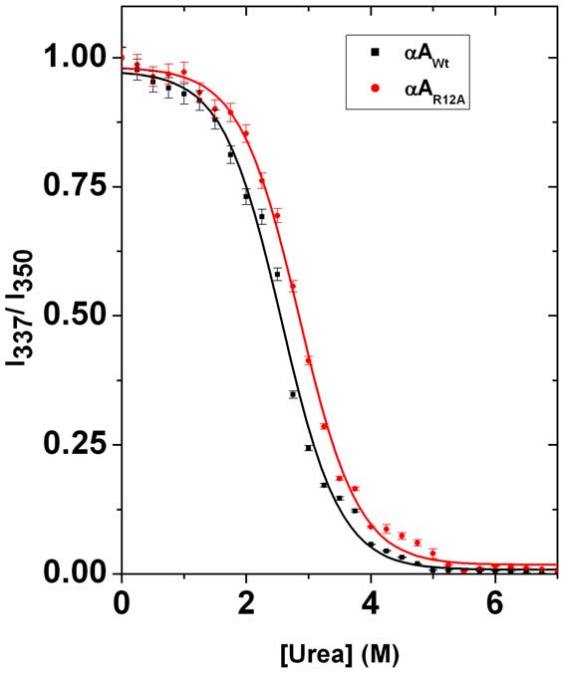
Thermodynamic stability of wild type and mutant (R12A) αA-crystallin. Equilibrium urea denaturation profile for 0.1 mg/ml wild type and mutant proteins at 25°C. The profile is normalized to a scale of 0 to 1. Symbols represent the experimental data points and the solid lines represent the best fit according to the three state model.

Several studies have revealed that other factors, such as oligomerization and structural perturbation, may also be required for the proper execution of the chaperone function of α-crystallin [15], [52], [53], [54], [57], [58]. To quantify the perturbation in the structural stability caused by the R12A mutation, we compared the thermodynamic stability of the Wt and mutant Hsp27 and αA- and αB-crystallin proteins. We measured the equilibrium unfolding caused by urea by following tryptophan fluorescence of the proteins at various urea concentrations. Tryptophan fluorescence intensities were recorded at 337 and 350 nm, respectively, at various concentrations of urea. The data were then plotted as the ratio of intensities at 337 and 350 nm as a function of the urea concentration (Fig. 7). A crude estimate of the transition midpoint (C_1/2_) from the sigmoidal analysis of the denaturation profiles revealed that the C_1/2_ value increased from 2.34 M of urea for Wt αA-crystallin to 2.72 M of urea for the R12A mutant (Fig. 7 and Table 5). This increase in the C_1/2_ value clearly indicated that the substitution of R12 by alanine increased the thermodynamic stability of αA-crystallin. The C_1/2_ value did not change in αB_R12A_ and Hsp27_R12A_ compared to the respective Wt proteins (Table 5). These results clearly indicated that the R12 residue had a marginal influence on the structural stability of αB-crystallin and Hsp27. To quantify the stability against chemical denaturation, all of the profiles were analyzed using a global three-state fitting procedure, according to the following equation:
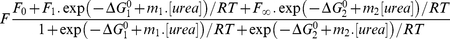
where F_0_, F_1_ and F_∞_ are the signal intensities for the 100% native, the 100% intermediate and the 100% unfolded forms, respectively. ΔG_1_^0^ refers to the standard free energy change between the native and the intermediate form, and ΔG_2_^0^ refers to the standard free energy change between the intermediate and the unfolded form. ΔG^0^, being the sum of ΔG_1_^0^ and ΔG_2_^0^, refers to the standard free energy change of unfolding (between the native and the unfolded form) at a urea concentration of zero. The fitted parameters are listed in Table 5. The standard free energy change of αA-crystallin unfolding at 25°C is 20.90 kJ/mol. This value of ΔG^0^ is comparable to that we and others have previously reported [42], [59]. The ΔG^0^value for αA_R12A_ increased to 25.54 kJ/mol, indicating an enhancement in thermodynamic stability by ∼4.5 kJ/mol. However, the R12A substitution had no effect on the structural stability of the other two small heat shock proteins (Table 5). In several previous studies, investigators found that increased chaperone function of α-crystallin was often associated with the greater structural stability of this protein [42], [58]. Therefore, we can also conclude that structural perturbation of αA-crystallin due to the R12A mutation is a cause for the enhancement of its chaperone function.

**Table 5:** The C_1/2_ and the ΔG^0^ values of the wild-type and R12A mutants of α-crystallin and Hsp27 at 25°C.

Proteins	C_1/2_ (M)	ΔG^0^ (kJ/mole)
αA_Wt_	2.34	20.90±0.62
αA_R12A_	2.72	25.54±0.66
αB_Wt_	2.41	22.44±0.21
αB_R12A_	2.43	22.34±0.78
Hsp27_Wt_	2.38	21.37±0.44
Hsp27_R12A_	2.36	21.38±0.56

In summary, our study showed that the molecular basis behind MGO-induced enhancement in the chaperone function of small heat shock proteins is different. Although mild MGO modification changes the conserved arginine residue (R12) in all three small heat shock proteins, this modification is likely beneficial only for αA-crystallin. Because αA-crystallin is predominantly found in the eye lens, MGO-induced enhancement in the chaperone function of this protein may be important in maintaining the transparency of the lens.

## Materials and Methods

Citrate synthase (CS), lactate dehydrogenase (LDH), dithiothreitol (DTT), lysozyme and bovine insulin were obtained from Sigma-Aldrich Chemical Co., LLC (St. Louis, MO, USA). CS was dialyzed in 40 mM HEPES buffer, pH 7.4, for 24 hr before use. 2-(p-toluidino) naphthalene-6-sulfonate (TNS) was obtained from Molecular Probes (Invitrogen, Carlsbad, CA, USA). Bovine γ-crystallin were purified from lenses, as previously described [60]. All other chemicals were of analytical grade.

### Modification of proteins by MGO

Small heat shock proteins (1.0 mg/ml Hsp27 and αA- and αB-crystallin) were incubated with either 2, 5 or 10 µM MGO (in 100 mM sodium phosphate buffer, pH 7.4) for 3 days at 37°C. The incubated samples were subjected to SDS-PAGE (12% gel) under reducing conditions.

### Identification of hydroimidazolone by mass spectroscopy

Gel pieces containing αA-crystallin, αB-crystallin and Hsp27 cut from the SDS-PAGE were first destained with 50% acetonitrile in 100 mM ammonium bicarbonate, and then 100% acetonitrile. Then, the proteins were reduced by 20 mM DTT at room temperature for 60 min, followed by the alkylation of the proteins using 50 mM iodoacetamide for 30 min in the dark. The reaction reagents were removed, and the gel pieces were washed with 100 mM ammonium bicarbonate and dehydrated in acetonitrile. Sequencing grade modified trypsin (Promega, Madison, WI) in 50 mM ammonium bicarbonate was added to the dried gel pieces and incubated at 37°C overnight. Proteolytic peptides extracted from the gel with 50% acetonitrile in 5% formic acid were then dried and dissolved in 0.1% formic acid. Liquid chromatography-tandem mass spectrometric analysis of the resulting peptides was performed on a LTQ Orbitrap Velos (Thermo Fisher Scientific, Waltham, MA) equipped with nanoACQUITY UPLC (Waters, Milford, MA) system. The spectra were acquired by data dependent methods consisting of a full scan and MS/MS on the ten most abundant precursor ions at the normalized collision energy of 35%. The data that were obtained were submitted to Mascot Daemon (Matrix Science, Boston, MA) for identification of the hydroimidazolone modification on arginine residues. The modification sites were then verified by manual interpretation of the MS/MS spectra.

### Cloning and purification of proteins

The previously described constructs for Wt αA- and αB-crystallin were used as templates [32], [58]. Hsp27 cDNA from Thermo Scientific Open Biosystems, Huntsville, AL was used as a template. Wild-type heat shock proteinsαA-, αB-crystallin and Hsp27 were amplified by PCR using the following primers.

αA FP: 5-GGCCATATGGACGTGACCATCCAGCAC

αARP: 3-CCCAAGCTTGGACGAGGGAGCCGAGGTG

αB FP: 5-GGCCATATGGACATCGCCATCCACCAC

αB RP: 3-CCCAAGCTTTTTCTTGGGGGCTGCGGTGAC

Hsp27 FP: 5- GGCCATATGACCGAGCGCCGCGTCCCCTTCTCG

Hsp27 RP: 3- CCCAAGCTTTTACTTGGCGGCAGTCTCATCGGATTT

The amplified PCR product was cloned into the pET23a vector using NdeI and HindIII restriction sites. The R12A mutants were generated by site-directed mutagenesis using the respective Wt cDNA as the template. The following primers were used.

αA_R12A_FP:5-TGGTTCAAGGCCACCCTGGGG

αA_R12A_RP:3-CCCCAGGGTGGCCTTGAACAA

αB_R12A_FP:5-TGGATCCGCGCCCCCTTCTTT

αB_R12A_RP:3-AAAGAAGGGGGCGCGGATCCA

Hsp27_R12A_ FP:5-TCGCTCCTGCGGGGCCCCAGC

Hsp27_R12A_ RP:3-GCTGGGGCCCCGCAGGAGCGA

The resulting PCR product was digested with DpnI and then transformed into *E.coli* DH5alpha cells. Plasmids from the resulting colonies were sequenced to confirm the presence of the mutation. The recombinant proteins were overexpressed in *E.coli* BL21(DE3) by induction with 250 µM IPTG when the OD_600 nm_ of the culture in LB broth reached ∼0.6. The bacterial pellet obtained after centrifugation at 10,000 g was suspended in 50 mM Tris, pH 8.0 containing 50 mM NaCl, 2 mM EDTA and 10 µl/ml of a protease inhibitor cocktail (Sigma). Lysozyme was added at 0.3 mg/ml to the cell suspension and incubated for 10 min at 37°C, followed by sonication on ice at 40 duty cycles at 30% amplitude. Benzonase nuclease (1.0 µl) was then added to the resulting cell lysate and incubated at 37°C in a shaker for 20 min, which was followed by the addition of sodium deoxycholate at 1.0 mg/ml and a subsequent incubation for 10 min at 37°C. DTT was then added to the lysate at a 5 mM concentration and incubated for 10 min at 37°C. The cell lysate was then centrifuged at 20,000 g for 30 min at 4°C. DNA in the lysate was precipitated by adding 0.2% polyethyleneimine followed by centrifugation at 20,000 g for 15 min. Ammonium sulfate was added to the lysate to reach 70% saturation, and the suspension was then left at 4°C overnight and then centrifuged at 20,000 g for 5 min. The resulting pellet was suspended in 50 mM sodium phosphate buffer (pH 7.4), which contained 150 mM NaCl and 5 mM DTT, and was then centrifuged at 20,000 g for 5 min. The supernatant was filtered with a 0.45 µm filter and applied onto a Superdex-200 prep grade (GE Healthcare, WI) gel filtration column that was pre-equilibrated with 50 mM sodium phosphate buffer pH 7.4. Fractions of 2.0 ml were collected and their OD_280 nm_ was recorded. The peak fractions were pooled and dialyzed overnight at 4°Cin 20 mM Tris, pH 8.0 that contained 0.1 mM EDTA. The dialyzed sample was applied onto a Q-Sepharose (GE Healthcare, WI) anion exchange column equilibrated with 20 mM Tris, pH 8.0 with 0.1 mM EDTA. The bound protein was eluted with a 0–1 M NaCl gradient. The protein peak fractions were pooled and dialyzed in PBS containing 0.1 mM EDTA and stored in aliquots at −80°C.

### Determination of molecular mass by multi-angle light scattering

The molar mass and the hydrodynamic radius of wild-type and R12A mutants of α-crystallin and Hsp27 were estimated by multi-angle light scattering measurements as previously described [32], [58]. The molar mass (M_w_) and the hydrodynamic radius (R_h_) of Wt and mutant proteins were determined using ASTRA (5.3.4) software developed by Wyatt Technology Corp.

### Determination of secondary and tertiary structure by CD spectroscopy

The far-UV CD spectra were measured at 25°C using a Jasco 815 spectropolarimeter (Jasco, Inc., Japan). The spectra were collected from 250 to 200 nm using a cylindrical quartz cell of 2 mm path length. Proteins (0.2 mg/ml) were dissolved in 10 mM phosphate buffer (pH 7.2). The resultant spectra after five scans were analyzed for secondary structure by the curve-fitting program CONTINLL [61].

The near-UV CD spectra were measured at 25°C using the same spectropolarimeter as stated above. The spectra were measured with a 0.5 mg/ml protein solution in 50 mM phosphate buffer (pH 7.2). The reported spectra were the average of 5 scans.

### Tryptophan fluorescence measurements

The intrinsic tryptophan fluorescence spectra of proteins (0.1 mg/ml) in 50 mM phosphate buffer (pH 7.2) at 25°C were recorded using a Fluoromax-4P spectrofluorometer (Horiba Jobin Mayer, USA). The excitation wavelength was set to 295 nm, and the emission spectra were recorded between 310 and 400 nm. Data were collected at a 0.5 nm wavelength resolution.

### Estimation of Surface hydrophobicity

The surface hydrophobicity of the different protein solutions (0.1 mg/ml of the wild-type and R12A mutants) was measured using a hydrophobic probe, TNS (emission: 350–520 nm, excitation: 320 nm), as previously described [33]. The concentration of TNS that was used was 100 µM.

### Chaperone assays

The chaperone assays were carried out as previously described [62]. The ratios (w/w) of αA-crystallin to CS, γ-crystallin and LDH were 1∶10, 1∶12 and 1∶28, respectively. The ratios (w/w) of αB-crystallin to CS, γ-crystallin and LDH were 1∶4, 1∶15 and 1∶28, respectively. The ratios (w/w) of Hsp27 to CS, γ-crystallin and LDH were 1∶10, 1∶37 and 1∶28, respectively.

### Equilibrium binding study

The chaperone-substrate binding study was performed by a membrane filtration method that we recently described [58]. Briefly, wild-type or mutant Hsp27 and αA- and αB-crystallin (12.5 µM) were incubated at 60°C for 1 hr with 2–18 µM γ-crystallin in 50 mM phosphate buffer containing 100 mM NaCl (pH 7.2). After equilibration, the incubation mixture was spun through a Microcon centrifugal device (4,000 g) fitted with a 100-kDa cut off membrane filter to separate the unbound substrate. The number of binding sites (n) and dissociation constant (K_d_) were determined by a similar procedure [33], [42], [58].

### Determination of structural stability of proteins

The structural stability of Wt and mutant proteins was determined by equilibrium chemical denaturation experiment. Wt and mutant proteins (0.05 mg/ml in 50 mM phosphate buffer, pH 7.5) were incubated separately with various urea concentrations (0–7 M) for 18 hrs at 25°C. Tryptophan fluorescence spectra of all samples were taken in the 300–400 nm region using 295 nm as the excitation wavelength. The equilibrium unfolding profile was fitted according to a three state model [42], [53], [58].
